# Predictors of Surgical Intervention in Dialysis Patients With Infective Endocarditis

**DOI:** 10.1093/ofid/ofy265

**Published:** 2018-10-20

**Authors:** John A Woller III, Victoria L Walsh, Chad Robichaux, Vinod H Thourani, Jesse T Jacob

**Affiliations:** 1 Division of Infectious Diseases, Atlanta, Georgia; 2 Department of Medicine, Atlanta, Georgia; 3 Emory University School of Medicine, Atlanta, Georgia; 4 Department of Cardiac Surgery, MedStar Heart & Vascular Institute, MedStar Health, Columbia, Maryland; 5 Division of Cardiothoracic Surgery, Department of Surgery, Emory University School of Medicine, Atlanta, Georgia

**Keywords:** end-stage renal disease, infective endocarditis, patient selection, surgical indications, valve surgery

## Abstract

**Background:**

The use of valve surgery for infective endocarditis (IE) in end-stage renal disease (ESRD) patients may be different than in the general population. We assessed predictors of early surgery in ESRD patients with IE.

**Methods:**

We conducted a retrospective cohort study among dialysis patients with left-sided IE between 2005 and 2015. Indications for surgery were based on current endocarditis guidelines. Patients were categorized as early valve replacement surgery or delayed/no surgery. We used logistic regression to determine independent predictors of early surgery.

**Results:**

Among 229 patients, 67 (29.3%) underwent early surgery. New congestive heart failure was the only high level of evidence indication independently associated with early surgery (odds ratio [OR], 12.1; 95% confidence interval [CI], 3.4–43.6). Transfer from outside hospital (OR, 5.4; 95% CI, 2.2–13.3), valve rupture (OR, 6.9; 95% CI, 2.6–17.9), coagulase-negative staphylococcus etiology (OR, 3.8; 95% CI, 1.4–10.6), and presence of any low level of evidence indication (OR, 5.9; 95% CI, 2.2–15.5) predicted early surgery. Preexisting valve disease (OR, 0.31; 95% CI, 0.12–0.82) and surgical contraindications (OR, 0.05; 95% CI, 0.005–0.4) predicted nonsurgical treatment.

**Conclusions:**

Among ESRD patients with IE, most surgical indications are not predictive of early surgery.

Patients receiving dialysis are at increased risk for infective endocarditis (IE) from the combination of transient bacteremia resulting from repeated vascular access, accelerated valvular calcification, and immune dysfunction [1–4]. It has been estimated that 3% of patients receiving hemodialysis will develop IE in their lifetime [5]. With a high morbidity and mortality in the general population, IE carries an even grimmer prognosis for end-stage renal disease (ESRD) patients. One-year survival rates for patients with both ESRD and IE are estimated to be 50% [6, 7], whereas the expected survival rate at 5 years with ESRD alone is 50% [6].

The 2015 American Heart Association (AHA) IE guideline provides antibiotic recommendations and indications for surgical intervention for all patients, based on clinical, microbiologic, and echocardiographic assessment [8]. These indications are divided into Class I (evidence or general agreement that surgery is useful and effective, generally focused on hemodynamic complications or poor response to medical therapy) and Class II (conflicting evidence and/or divergence of opinion regarding whether surgery is useful and/or effective, mostly focused on preventing embolic consequences of IE). The benefit of reducing long-term mortality may outweigh the significant risks of surgery in many ESRD patients, particularly among those with guideline-based indications [9–14]. Patients who meet established guidelines for surgical treatment of IE may not be those who actually go to the operating room. Because ESRD patients are often considered high risk, surgery may be deferred to optimize medical status in acutely ill patients in spite of clear surgical indications but may be performed sooner on less ill patients [11, 14, 15]. Recent data suggest that, despite overall high risk, patients with ESRD likely benefit from appropriate surgical intervention [16]. We sought to determine whether, among patients receiving dialysis with left-sided IE, having an indication for surgery was associated with undergoing valve replacement surgery.

## METHODS

In this retrospective cohort study, all patients receiving chronic dialysis hospitalized with IE from 2005 to 2015 at two 500-bed academic hospitals, both regional referral centers for the medical and surgical care of IE, were eligible for inclusion. Patients were initially identified using the presence of ICD-9 codes for ESRD (585.6) and IE (421.0, 421.1, 421.9) during the same encounter, and charts were manually reviewed to confirm that patients were receiving dialysis for at least 30 days before the diagnosis of IE and met the modified Duke criteria for definite IE [17] with evidence of mitral or aortic valve involvement. Patients with right-sided or both left- and right-sided IE were excluded because most of the Class I and II indications deal with left-sided endocarditis. Data were extracted using a structured chart review of the clinical notes, laboratory and radiology results, and electrocardiogram (ECG) and echocardiography results and were supplemented by an existing data set spanning 1992–2012.

The indications for surgery from the 2015 AHA Endocarditis Guidelines (Table 1) were used [8]. Patients were followed through death, discharge to hospice, or loss to follow-up as of October 28, 2016. We defined “early surgical intervention” as valve replacement or repair within 6 weeks of the diagnosis of IE and during the index hospitalization; no early surgical intervention included patients who never underwent surgery or had surgery more than 6 weeks after the diagnosis of IE and/or after index hospitalization. We assessed any documented intravenous (IV) drug use in the 6 months before hospitalization. Vegetations were considered large if ≥10 mm [18]. Persistence (such as for fever or positive blood cultures) was determined if present after 7 days of effective therapy. Effective therapy was considered to be any antibiotic active against the pathogen, even if not the optimal choice, such as vancomycin for methicillin-sensitive *Staphylococcus aureus* (MSSA). Enterococci or methicillin-resistant *Staphylococcus aureus* (MRSA) resistant to vancomycin, gram-negative organisms resistant to ≥3 antibiotic Classes, and fungi were considered difficult-to-treat organisms.

**Table 1.** **Indications and Contraindications for Surgery Based on the 2015 AHA Endocarditis Guidelines** [8]

Class I Indications for Surgery
New congestive heart failure – new-onset New York Heart Association Class III or IV CHF refractory to medical therapy not present before the diagnosis of IE (including if the patient had a lower Class of heart failure before diagnosis)
Abscess or fistula visualized via echocardiogram
New conduction delay – any new AV nodal block or bundle branch block compared with prior ECG)
Difficult-to-treat organism
Persistent infection – fever or positive blood culture despite at least 7 days of effective antibiotic therapy
Class IIa Indications
Large vegetation with new valve regurgitation
Recurrent emboli while on effective antimicrobial therapy and evidence of persistent or enlarging vegetation on repeat echocardiogram
Relapsing prosthetic valve IE – evidence of IE that has either recurred or failed to resolve within 6 months of the initial diagnosis of IE
Recurrent emboli while on effective antimicrobial therapy with IE of a prosthetic valve
Class IIb Indication
Large vegetation, particularly when attached to the anterior leaflet of the mitral valve
Contraindications
Recent or ongoing intravenous drug use
Intracranial hemorrhage
Severe neurological damage (eg, coma)

Abbreviations: AHA, American Heart Association; AV, aortic valve; CHF, congestive heart failure; IE, infective endocarditis; ECG, electrocardiogram.

Chronic corticosteroid use was defined as any dose of oral steroids given for at least 30 days before hospitalization. Severe neurological damage was defined as coma or quadriplegia. Other infections were those distinct from sequelae of endocarditis.

The primary outcome was early surgical intervention. Secondary outcomes were in-hospital mortality, including discharge to hospice. The Emory University Institutional Review Board approved this study.

### Statistical Analysis

SAS, version 9.4 (SAS Institute, Cary, NC), was used for statistical analysis. Categorical variables were expressed as a number and percentage, and continuous variables as a median with first and third quartiles. Characteristics of patients who underwent early surgical intervention were compared with those of patients who did not undergo early surgical intervention. Categorical variables were analyzed using the χ^2^ or Fisher exact test, as appropriate, and continuous variables were analyzed using the Wilcoxon rank-sum test. Bivariate analysis was conducted to identify characteristics that were independently associated with early surgery. All variables that were significantly associated with early surgery at the α = 0.05 level were included in the full multivariate model. Backwards model selection with a cut-point of α = 0.05 was conducted to identify a final multivariate model. Age at diagnosis was identified as an a priori predictor of early surgery and was thus forced into the final model.

A post hoc sensitivity analysis was performed excluding patients transferred from outside hospitals. In-hospital mortality was compared using the χ^2^ test.

## RESULTS

### Overall Cohort Characteristics

**Figure 1.**
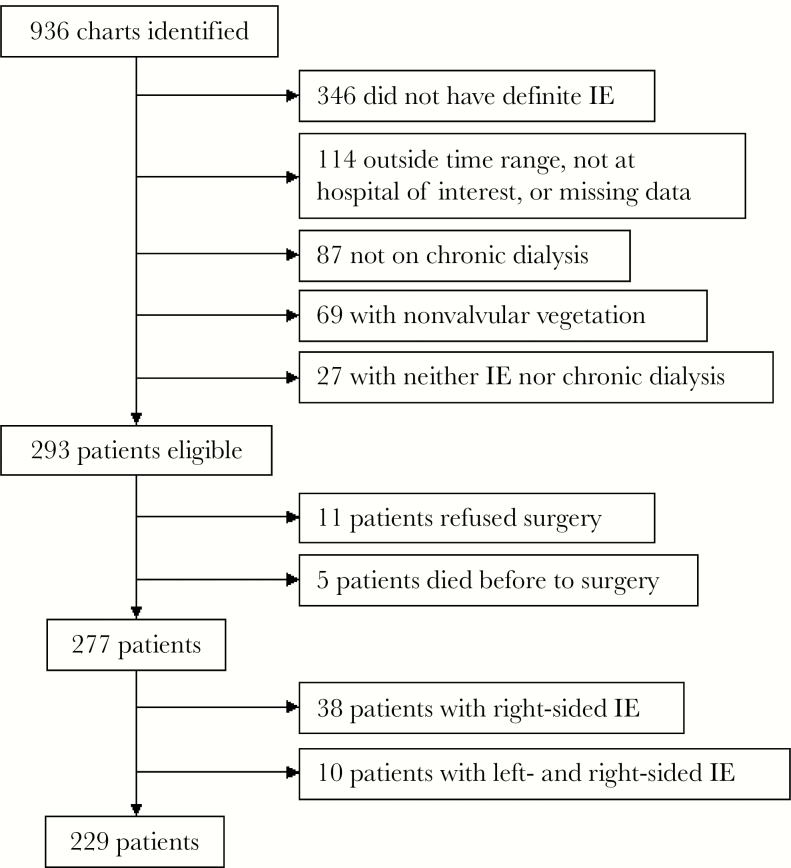
Patients excluded from study. Abbreviation: IE, infective endocarditis.

Out of 936 patients identified using ICD-9 codes, 229 were included in the final study (Figure 1). Eleven patients refused surgery, and 5 died before a planned operation and were excluded from analysis. The median age was 57 years (Table 2). Most patients were male (51.1%), black (85.7%), nondiabetic (51.5%), and used a catheter for hemodialysis (HD; 52.3%); peritoneal dialysis was rare (4.1%). Sixty-seven patients (29.3%) underwent early surgery, and an additional 10 (4.4%) underwent delayed valve replacement surgery after discharge, 2 of whom underwent surgery within 6 weeks of diagnosis.

**Table 2.** Baseline Characteristics of Patients With End-Stage Renal Disease on Dialysis Hospitalized With Infective Endocarditis, Stratified by Early Surgery

	Overall (n = 229)	Early Surgery (n = 67)	No Early Surgery (n = 162)	*P* Value
	No. (%) or Median (25th, 75th Percentile)			
Age, y	57 (48, 65)	53 (44, 61)	57 (49, 67)	.02
Female sex	112 (48.9)	37 (55.2)	75 (46.3)	.22
Black race (n = 223)	191 (85.7)	53 (84.1)	138 (86.3)	.68
Diabetes mellitus	111 (48.5)	33 (49.3)	78 (48.2)	.88
HIV infection	16 (7.0)	0 (0)	16 (9.9)	.004
Chronic corticosteroid use	15 (6.6)	5 (7.5)	10 (6.2)	.77
IV drug use (n = 212)	2 (0.9)	0 (0)	2 (1.3)	1.0
History of IE (227)	14 (6.2)	6 (9.0)	8 (5.0)	.26
Existing valve disease (n = 224)	102 (45.5)	19 (29.7)	83 (51.9)	.003
Prosthetic valve	26 (11.4)	7 (10.5)	19 (11.7)	.78
OSH transfer	77 (33.8)	40 (59.7)	37 (23.0)	<.0001
Dialysis access (n = 220)				
IV catheter	115 (52.3)	32 (50.0)	83 (53.2)	.67
Other access	105 (47.7)	32 (50.0)	73 (46.8)	
Time since dialysis initiation (n = 197), mo	37 (13, 75)	18 (11, 60)	42.5 (14.5, 80)	.03
Duration of dialysis access (n = 164), wk	32 (11, 86.5)	28.5 (11, 64)	35 (11, 89)	.32

Abbreviations: IV, intravenous; IE, infective endocarditis; OSH, outside hospital.

Few patients (51, 24.4%) had documented fever at the time of diagnosis (Table 3). Many patients (92, 40.2%) had clinical or radiographic evidence of embolic phenomena, with stroke being the most common (55, 24.1%). The majority of patients had a vegetation visualized on echocardiography (210, 91.7%) or new valve regurgitation (136, 59.9%). Most patients (155, 67.7%) had mitral valve IE, followed by aortic valve IE (98, 42.8%), with few (24, 10.5%) having both aortic and mitral involvement. Gram-positive cocci were the most common organisms (201, 87.8%), including 104 (45.4%) due to *S. aureus,* with slightly over half (53/104, 51%) being methicillin-resistant. Fifty-three cases (23.1%) were due to coagulase-negative staphylococci (Table 4).

**Table 3.** Clinical, Laboratory, and Echocardiographic Characteristics of Patients With End-Stage Renal Disease on Dialysis Hospitalized With Infective Endocarditis, Stratified by Early Surgery

	Overall (n = 229)	Early Surgery (n = 67)	No Early Surgery (n = 162)	*P* Value
	No. (%) or Median (25th, 75th Percentile)			
Altered mental status (n = 227)	67 (29.5)	12 (17.9)	55 (34.4)	.01
Any vascular phenomena	92 (40.2)	28 (41.8)	64 (39.5)	.75
Cerebrovascular accident (n = 228)	55 (24.12)	12 (17.9)	43 (26.7)	.16
Pulmonary embolus (n = 228)	9 (4.0)	3 (4.5)	6 (3.7)	.79
Intracranial hemorrhage	15 (6.6)	1 (1.5)	14 (8.6)	.07
Severe neurological damage (n = 225)	18 (8)	1 (1.5)	17 (10.6)	.03
Concurrent infection	63 (27.5)	11 (16.4)	52 (32.1)	.02
New-onset congestive heart failure	31 (13.6)	25 (37.3)	6 (3.7)	<.0001
Involved valve				
Aortic	98 (42.8)	38 (56.7)	60 (37.0)	.006
Mitral	155 (67.7)	43 (64.2)	112 (69.1)	.47
New valvular regurgitation (n = 227)	136 (59.9)	57 (87.7)	79 (48.8)	<.0001
Intracardiac abscess or fistula (n = 219)	31 (14.2)	20 (32.3)	11 (7.0)	<.0001
Valvular vegetation	210 (91.7)	63 (94.0)	147 (90.7)	.60
Large vegetation (n = 145)	84 (57.9)	33 (76.7)	51 (50.0)	.003
Valve rupture (n = 220)	66 (30)	40 (61.5)	26 (16.8)	<.0001
Fever on admission (n = 209)	51 (24.4)	10 (19.6)	41 (26.8)	.18
Laboratory values				
Hematocrit	31.1 (27.5, 35.1)	30.2 (27.3, 33.1)	31.4 (27.5, 35.9)	.19
White blood cell count × 1000/mm^3^	11.3 (8.6, 16.0)	12.1 (8.9, 16.1)	11.2 (8.4, 16.0)	.55
Platelet count × 1000/mm^3^	212 (155, 273)	232 (190, 322)	198.5 (137, 263)	.002
Glucose, mg/dL	112 (90, 147)	109 (91, 137)	113.5 (87, 149)	.88
Albumin, g/dL	2.6 (2.2, 3.0)	2.6 (2.2, 2.9)	2.7 (2.2, 3.1)	.42
Blood urea nitrogen, mg/dL	42 (30, 64)	37 (28, 52)	46 (31, 67)	.03

**Table 4.** Microbiological Characteristics of Patients With End-Stage Renal Disease on Dialysis Hospitalized With Infective Endocarditis, Stratified by Early Surgery

	Overall (n = 229)	Early Surgery (n = 67)	No Early Surgery (n = 162)	*P* Value
	No. (%)			
Gram-positive cocci	201 (87.8)	57 (85.1)	144 (88.9)	.42
*Staphylococcus aureus*	104 (45.4)	23 (34.3)	81 (50.0)	.03
MSSA	53 (23.1)	11 (16.4)	42 (25.9)	.12
MRSA	51 (22.3)	12 (17.9)	39 (24.1)	.31
VISA	3 (1.3)	3 (4.5)	0 (0)	.02
Coagulase-negative staphylococci	53 (23.1)	27 (40.3)	26 (16.1)	<.0001
Methicillin resistant	26 (11.4)	13 (19.4)	13 (8.0)	.01
Streptococci	7 (3.1)	2 (3.0)	5 (3.1)	1.0
Enterococci	37 (16.2)	5 (7.5)	32 (19.8)	.03
VRE	7 (3.1)	0 (0)	7 (4.3)	.11
Gram-negative rods	2 (0.9)	1 (1.5)	1 (0.6)	.50
ESBL-producing GNR	1 (0)	0 (0)	1 (0.6)	1.0
*Corynebacterium jeikeium*	1 (0.4)	1 (1.5)	0 (0)	.59
Fungi	2 (0.9)	0 (0)	2 (1.2)	1.0
No growth	23 (10.4)	8 (11.9)	15 (9.3)	.54
Difficult-to-treat organism	13 (5.7)	3 (4.5)	10 (6.2)	.76
Persistently positive blood cultures (n = 223)	12 (5.4)	2 (3.1)	10 (6.3)	.52
Persistent fever (n = 199)	22 (11.1)	6 (10.7)	16 (11.2)	.92

Abbreviations: ESBL, extended-spectrum beta-lactamase; GNR, Gram-negative rods; MRSA, methicillin-resistant *Staphylococcus aureus*; MSSA, methicillin-sensitive *S. aureus*; VISA, vancomycin-intermediate *S. aureus*; VRE, vancomycin-resistant enterococci.

Most patients (127, 55.5%) had a documented Class I or Class IIa indication for surgery (Table 5). Nearly half (95, 46.3%) had a Class I indication; 52 (22.7%) had a Class I indication for surgery and did not receive early surgical intervention. Out of these, 12/52 (23.1%) had a documented contraindication to surgery. An additional 20 patients (8.7%) had a Class IIa indication and did not receive early surgery, of whom 7 (35%) had a contraindication to surgery.

**Table 5.** Frequency of Indications for Surgery Among Patients With End-Stage Renal Disease on Dialysis Hospitalized With Infective Endocarditis, Stratified by Early Surgery

	Overall (n = 229)	Early Surgery (n = 67)	No Early Surgery (n = 162)	*P* Value
	No. (%)			
Any Class I indication (n = 205)	95 (46.3)	43 (70.5)	52 (36.1)	<.0001
New-onset CHF (n = 228)	31 (13.6)	25 (37.3)	6 (3.7)	<.0001
New conduction delay (n = 210)	29 (13.8)	10 (17.2)	19 (12.5)	.37
Abscess or fistula on echocardiogram (n = 219)	31 (14.2)	20 (32.3)	11 (7.0)	<.0001
Persistent infection (n = 197)	29 (14.7)	7 (12.7)	22 (15.5)	.62
Difficult-to-treat organism	13 (5.7)	3 (4.5)	10 (6.2)	.76
Any Class IIa indication	70 (30.6)	34 (50.8)	36 (22.2)	<.0001
Recurrent emboli with persistent vegetation (n = 216)	12 (5.6)	4 (6.4)	8 (5.2)	.75
Valve regurgitation and mobile vegetation >10 mm (n = 227)	57 (25.1)	28 (43.1)	29 (17.9)	<.0001
Prosthetic valve IE with recurrent emboli (n = 224)	2 (0.9)	0 (0)	2 (1.3)	1.0
Relapsing prosthetic valve IE (n = 226)	7 (3.1)	5 (7.6)	2 (1.3)	.02
Class IIB indication				
Mobile vegetation >10 mm (n = 145)	84 (57.9)	33 (76.7)	51 (50.0)	.003
Any contraindication	28 (12.2)	2 (3.0)	26 (16.0)	.007

Abbreviations: CHF, congestive heart failure; IE, infective endocarditis.

### Predictors of Early Surgery

Clinical and demographic factors significantly associated with early surgical intervention in bivariate analysis included any surgical indication (Class I or II) and specific Class I (new congestive heart failure [CHF] and abscess or fistula visualized via echocardiogram) and Class II (large vegetation, new valve regurgitation) indications, outside hospital (OSH) transfer, aortic valve involvement, new valve regurgitation, large vegetation, coagulase-negative staphylococci, valve rupture, higher platelet count, and multivalve disease (Table 8). Factors associated with not undergoing early surgery in bivariate analysis were age >65 years, preexisting valve disease, altered mental status (AMS) at diagnosis, *S. aureus* etiology, and concurrent infection.

In multivariate analysis, OSH transfer (odds ratio [OR], 5.4; 95% confidence interval [CI], 2.2–13.3), coagulase-negative staphylococci infection (OR, 3.8; 95% CI, 1.4–10.6), valve rupture (OR, 6.9; 95% CI, 2.6–17.9), new CHF (OR, 12.1; 95% CI, 3.6–43.6), and the presence of any Class IIa indication (OR, 5.9; 95% CI, 2.2–15.5) predicted early surgery (Table 9). Class I indications other than new CHF, the aggregate variable for any surgical indication, and the Class IIb indication (presence of a large vegetation) were not independently associated with early surgery. Preexisting valve disease (OR, 0.31; 95% CI, 0.12–0.82) and the presence of a contraindication (OR, 0.05; 95% CI, 0.01–0.4) were protective against early surgery.

Patients transferred from an OSH more commonly had diabetes mellitus (58.4% vs 43.1%; *P* = .03) and documentation of evaluation by cardiothoracic (CT) surgery (81.8% vs 51.3%; *P* < .0001) but were less likely to be black (72.6% vs 92.6%; *P* < .0001) or have HIV (0% vs 10.6%; *P* = .002) (Table 6). In a sensitivity analysis excluding transferred patients, no major differences in the independent predictors of early surgery were noted (Table 9).

**Table 6.** Characteristics of Patients With End-Stage Renal Disease on Dialysis Hospitalized With Infective Endocarditis, Stratified by Transfer From an Outside Hospital

	Overall (n = 228)	OSH Transfer (n = 77)	No OSH Transfer (n = 151)	*P* Value
	No. (%) or Median (25th, 75th Percentile)			
Age, y	57 (48, 65.5)	55 (47, 64)	57 (50, 66)	.30
Female sex	112 (48.9)	37 (48.1)	75 (48.7)	.60
Black race (n = 221)	191 (86.0)	53 (72.6)	138 (92.6)	<.0001
Diabetes mellitus	110 (48.3)	45 (58.4)	65 (43.1)	.03
HIV infection	16 (7.0)	0 (0)	16 (10.6)	.002
Chronic corticosteroid use	15 (6.6)	5 (6.5)	10 (6.6)	1.0
IV drug use (n = 210)	2 (0.9)	0 (0)	2 (1.4)	1.0
History of IE (n = 226)	14 (6.2)	6 (8)	8 (5.3)	.42
Existing valve disease (n = 223)	102 (45.7)	27 (36.5)	75 (50.3)	.051
Prosthetic valve	26 (11.4)	12 (15.6)	14 (9.3)	.16
Dialysis access				
IV catheter	114 (52.1)	35 (49.3)	79 (53.4)	.57
Other access	105 (47.9)	36 (50.7)	69 (46.6)	
Time since dialysis initiation (n = 223), mo	37 (13, 75)	23.5 (9, 60.5)	39 (14, 79)	.089
Duration of dialysis access (n = 164), wk	32 (11, 86.5)	32 (15, 68)	35 (10, 89)	.76
Any Class I indication (n = 204)	95 (46.6)	40 (61.5)	55 (39.6)	.003
New CHF (n = 227)	31 (13.7)	17 (22.4)	14 (9.3)	.007
New conduction delay (n = 209)	29 (13.9)	7 (10.1)	22 (15.7)	.27
Abscess or fistula (n = 218)	31 (14.2)	17 (24.6)	14 (9.4)	.003
Difficult-to-treat organism	13 (5.7)	5 (6.5)	8 (5.3)	.77
Persistent infection (n = 196)	29 (14.8)	10 (16.7)	19 (14.0)	.62
Any Class IIa indication	69 (30.3)	33 (42.9)	36 (23.8)	0.003
Recurrent emboli with persistent vegetation (n = 215)	12 (5.6)	4 (5.7)	8 (5.5)	1.0
Valve regurgitation and mobile vegetation >10 mm (n = 227)	56 (24.8)	27 (35.5)	29 (19.3)	.008
Prosthetic valve IE with recurrent emboli (n = 223)	2 (0.9)	2 (2.7)	0 (0)	.11
Relapsing prosthetic valve IE (n = 225)	7 (3.1)	3 (4.0)	4 (2.7)	.69
Class IIb indication				
Mobile vegetation >10 mm (n = 144)	83 (57.6)	33 (76.7)	50 (49.5)	.003
Any contraindication	28 (12.3)	13 (16.9)	15 (9.9)	.13

Abbreviations: CHF, congestive heart failure; IE, infective endocarditis; IV, intravenous; OSH, outside hospital.

### Reasons for Delayed or No Surgery

In 75 of the 162 patients (46.3%) who did not undergo early valve replacement surgery, a reason for deferring surgery was documented in the chart (Table 7). In 10 cases (13.3%), clear contraindications (severe neurological damage or intracranial hemorrhage) were cited. Specific or general comorbidities were given as the reason for not operating in 12 cases (16%), with ESRD being specifically cited in 2 cases. The patient was considered a high-risk or poor surgical candidate in 24 cases (32%).

**Table 7.** Reasons Cited by Cardiothoracic Surgery Service for Not Offering Early Surgery

Reason Given	Frequency (n = 75), %
Poor surgical candidate (including poor functional status)	24 (32.0)
Lack of benefit/lack of indications	15 (20.0)
Comorbidities	12 (16.0)
Guideline-based contraindication	10 (13.3)
Persistent infection, or lack of complete antibiotic course	4 (5.3)
Presence of IV HD catheter	2 (2.7)
Medical clearance	2 (2.7)
Extensive calcification of aorta or mitral valve	2 (2.6)
Multiple prior sternotomies	1 (1.3)
Culture-negative IE	1 (1.3)
Stroke in operating room^a^	1 (1.3)
Anemia and refusal to receive blood products	1 (1.3)

Abbreviations: CHF, congestive heart failure; HD, hemodialysis; IE, infective endocarditis; IV, intravenous; OBGYN, obstetrics and gynecology.

^a^This patient was taken to the operating room and was thus counted as undergoing surgery for the purposes of surgical decision-making; however, the patient was considered not to have undergone surgery for survival analysis.

**Table 8.** Predictors of Surgical Intervention Among Patients With ESRD and Left-Sided IE in Bivariate Modeling

Variable	Odds Ratio	95% Confidence Interval	*P* Value
Age at diagnosis	0.97	0.94–0.99	.003
Outside hospital transfer	4.71	2.56–8.68	<.0001
Altered mental status	0.43	0.21–0.87	.02
Concurrent infection	0.37	0.18–0.78	.009
Aortic valve involvement	2.33	1.30–4.17	.004
New regurgitation	7.49	3.36–16.69	<.0001
Large vegetation	2.20	1.22–3.94	.008
Valve rupture	8.33	4.31–16.10	<.0001
Existing valve disease	0.37	0.20–0.68	.002
Multivalve disease	4.12	1.73–9.84	.001
*Staphylococcus aureus*	0.49	0.73–0.90	.02
Coagulase-negative staphylococci	3.65	1.91–6.96	<.0001
Methicillin-resistant	2.83	1.23–6.49	.01
Platelet count	1.004	1.001–1.006	.01
Any Class I indication	4.05	2.12–7.74	<.0001
New CHF	15.85	6.1–41.2	<.0001
Abscess or fistula	6.52	2.89–14.70	<.0001
Any Class IIa indication	3.41	1.86–6.24	<.0001
Large vegetation with recurrent emboli	3.47	1.84–6.55	.0001
Contraindication to surgery	0.16	0.04–0.72	.02

Abbreviations: CHF, congestive heart failure; ESRD, end-stage renal disease; IE, infective endocarditis.

**Table 9.** Variables Included in the Final Model to Assess Predictors of Surgical Intervention in ESRD Patients With Left-Sided IE

	Odds Ratio	95% Confidence Interval	Odds Ratio^a^	95% Confidence Interval^a^
Age at diagnosis	0.98	0.95–1.01	0.97	0.93–1.01
Existing valve disease	0.31	0.12–0.82	0.24	0.07–0.87
OSH transfer	5.4	2.2–13.3	-	-
Coagulase-negative staphylococci	3.8	1.4–10.6	5.6	1.4–22.4
Valve rupture	6.9	2.6–17.9	3.2	0.94–11.1
New CHF	12.1	3.4–43.6	6.8	1.3–34.7
Any Class IIa indication	5.9	2.2–15.5	6.2	1.8–21.9
Any contraindication	0.05	0.005–0.4	-	-

Fifteen patients were excluded from the final model due to missing data points; 214 patients are included in this model.

Abbreviations: CHF, congestive heart failure; ESRD, end-stage renal disease; IE, infective endocarditis; OSH, outside hospital.

^a^These columns represent a sensitivity analysis excluding patients transferred from outside hospitals. In this model, when the variable “any contraindication to surgery” was included, quasi-separation of data points was observed; this variable was therefore excluded.

**Figure 2.**
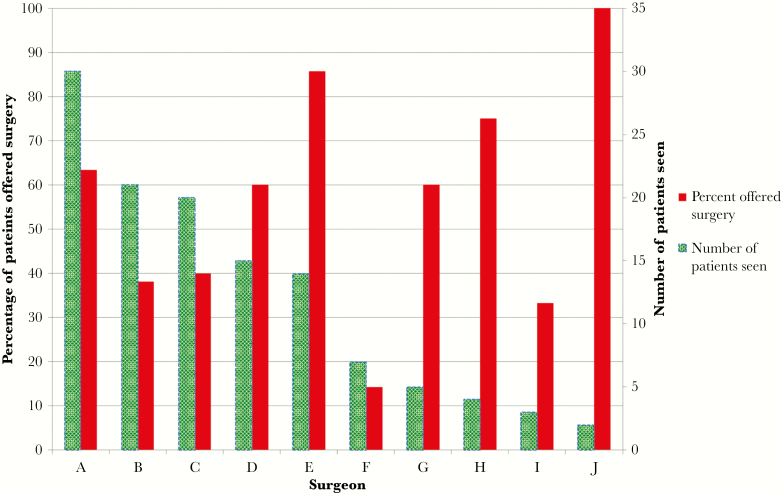
Comparison of the number of patients evaluated by each surgeon and the percentage of patients offered early surgical intervention.

Evidence of CT surgery evaluation was found in 141 (61.6%) patients. Of patients with documented surgical evaluation, 65/141 (46.1%) underwent early surgical intervention. In 121 cases, we identified the primary attending surgeon. In total, 10 CT surgeons (lettered A–K based on decreasing volume of patients evaluated) had surgical intervention rates from 14.3% (1/7) to 100% (2/2), with an overall rate of 54.5% (Figure 2). Patients evaluated by surgeon E were offered surgery (12/14, 85.7%) more frequently than those evaluated by the other 9 surgeons (54/107, 46.9%; *P* = .02). Of the 127 patients with any indication for surgery, there was evidence of CT surgery evaluation in 102 (80.3%).

**Figure 3.**
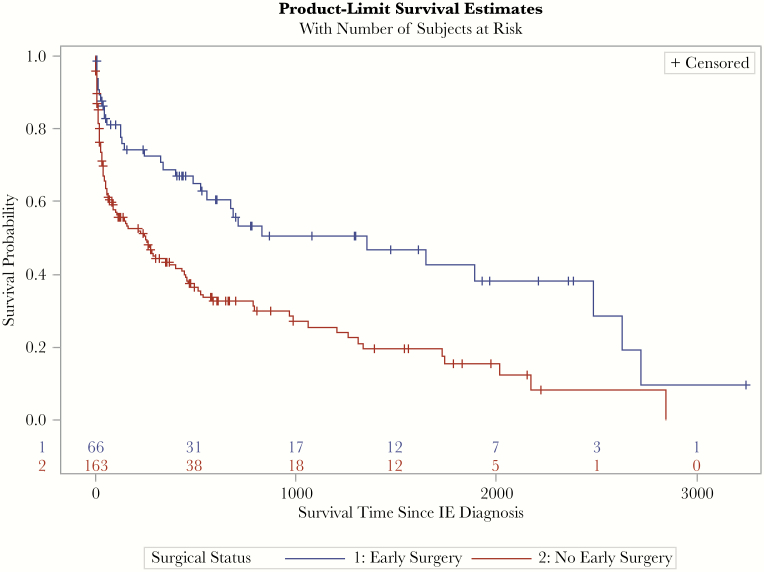
Overall survival stratified by patients who underwent early surgery and those who did not.

None of the 16 patients with HIV were offered early surgery (0% vs 9.9%; *P* = .004), though 2 subsequently underwent delayed valve replacement surgery. Seven HIV patients had Class I indications for surgery, including 1 with new CHF, and 1 had a guideline-based contraindication. Most (9/16, 56.3%) patients with HIV were taking antiretroviral therapy and had good immunologic (CD4 count >200) and virologic responses (undetectable viral load).

### In-Hospital Mortality and Survival Analysis

In-hospital mortality occurred in 51 (22.3%) patients and was similar in patients who underwent early surgery compared with those who did not (15.2% vs 25.2%; *P* = .10).

## DISCUSSION

In this large cohort of ESRD patients with IE, new-onset congestive heart failure refractory to medical therapy was the only Class I indication to independently predict early surgery, and was also its single strongest predictor (OR, 12.1; 95% CI, 3.6–43.6). All Class IIa indications for surgery (as a group), OSH transfer, valve rupture, and coagulase-negative staphylococci were also independently associated with early surgery. Importantly, no Class I indication other than CHF or individual Class IIa indication for surgery predicted surgical intervention, suggesting that many patients who may have benefited from surgery did not receive it. This may be due to contraindications, whether objectively based on guidelines or based on clinical assessment not clearly covered by the guidelines. The list of reasons for not offering surgery, though only available in the minority of patients, suggests that comorbidities and lack of benefit are frequent reasons, and it is possible that ESRD itself is a major consideration. Prosthetic valve endocarditis in ESRD patients carries a high mortality (Powell R, Steinberg JP, Jacob JT, manuscript in preparation). However, given the high mortality in these patients, a clear rationale for or against surgery should not only be discussed with patients, but also recorded in the chart.

The presence of a guideline-based contraindication and the presence of preexisting valve disease were associated with not having early surgery. Guideline-based contraindications to surgery are generally considered absolute, with a suggested delay of 4 weeks in the case of hemorrhagic stroke [8]. Many patients with ESRD have preexisting valve disease; the inverse association between existing valve disease and early surgery may explain in large part the low frequency of surgery. Greater understanding of how preexisting disease increases surgical risk is needed, especially when compared with medical therapy in patients with clear indications for surgery.

Transfer from an OSH independently predicted early surgery, a finding consistent with the existing literature [14]. Providers transferring patients from an OSH may have already communicated with a surgeon at the receiving hospital about potential risks and benefits; patients may thereby have been effectively screened as operative candidates. A higher percentage of transfer patients had evidence in the chart of evaluation by CT surgery, in part because they were often transferred directly to the CT surgical service. Some patients may have been transferred for the explicit purpose of undergoing valve replacement surgery. When using our final multivariate model for predictors of early surgery but excluding transferred patients, the results were similar, suggesting that our results may be generalizable to hospitals with fewer transferred patients.

In our study, infection with *S. aureus* was more common in the nonsurgical group, although it was not a predictor of early surgery. However, infection with coagulase-negative staphylococci, typically considered a less virulent organism, independently increased the likelihood of surgical intervention. A large prospective cohort study among non-ESRD patients also found *S. aureus* to be an independent predictor of nonsurgical treatment [14]. Observational data suggest that surgical intervention is associated with lower mortality in patients with IE due to *S. aureus* [9]. In our population, patients with *S. aureus* may have been more ill, increasing perceived risk of surgery.

None of the 16 patients with HIV underwent early surgery. HIV infection could not be included in our logistic regression model due to small sample size. From 1985 to 2013, there were no cases of patient-to-surgeon transmission of HIV, suggesting that the risk to providers is low [19]. Large retrospective studies have demonstrated that outcomes are similar or minimally different among HIV-infected and non-HIV-infected patients [20–22], and the 2005 AHA IE guidelines state that “HIV infection is not a contraindication for cardiac surgery, and postoperative complications, including mortality, are not increased in the HIV-infected population” [23]. Patients with HIV may be perceived as being at higher risk for surgery than the actual risk, leading to deferral of surgery.

Only 141 (61.6%) patients had documentation of surgical consultation. Some patients may have been evaluated using direct provider communication or telephone consultations that were not captured in the chart or documentation may have been missing. All patients with at least 1 indication for surgery should be considered for surgery, including some form of evaluation by CT surgery. However, among all patients with a Class I or Class IIa indication for surgery, 25/127 (19.7%) had no record of CT surgery evaluation. Despite high surgical risk, consultation with a CT surgery team may lead to improved shared decision-making by providers and a more informed discussion with the patient.

Overall, 29.3% of patients in our study underwent valve replacement surgery. This is slightly below the reported 31% of mostly non-ESRD patients in the United States who underwent valve replacement surgery between 1990 and 2010 [24]. Moderate to severe renal disease has been shown to be an independent predictor of nonsurgical treatment among all patients with IE [14]. Our data show that CT surgeons frequently do not offer surgery in this population, even in the presence of clear indications. Among patients with left-sided IE, 22.7% had a Class I indication for surgery and did not receive surgical treatment. In total, 31.4% had either a Class I or Class IIa indication and did not undergo early surgery. Many consultation notes described the patient as a “poor surgical candidate” (or similar description), which reflects both real and perceived high risk for surgery. Persistent infection is a Class I indication for surgery in the AHA guidelines, yet was cited as a reason not to operate in 4 cases. Notably, patients with active infection carry a higher risk of perioperative mortality [11]. However, patients who may benefit the most from surgery are often those at highest risk; the decision to operate must be individualized based on the clinical scenario [24].

In-hospital mortality was not significantly different between the surgery and no surgery groups. Without controlling for other patient characteristics, it is unclear whether surgery benefits patients with ESRD and IE. The effects of survivorship bias and selection bias in patients with IE undergoing surgery have been previously summarized in the 2015 AHA endocarditis guidelines [8, 13, 25].

This study has several strengths. Most IE studies include few patients with ESRD. This study is, to our knowledge, the largest assessment of left-sided IE in ESRD and is drawn from the largest cohort of patients with ESRD and IE. Although most studies assessing surgery in IE generalize the indications, such as “embolic event,” our approach used the precise definitions found in the 2015 AHA endocarditis guidelines, most of which contain multiple factors. Additionally, we examined Class I and Class II indications both in combination and separately to assess in more detail the adherence to these indications.

This study also has limitations. Some data were missing from the electronic medical record. Use of administrative billing data to identify patients may have missed some eligible patients. Both hospitals in our study are tertiary referral centers for valve surgery, and our study population may not be generalizable to other facilities.

In this population of patients with ESRD and IE, most established surgical indications are not independently associated with early surgery. Nearly one-fourth of patients with left-sided IE, most of whom were evaluated by the CT surgery service, had a Class I indication for surgery and were not offered early surgery, reflecting the complexity of caring for this population with multiple comorbidities and high acuity of illness. More prospective data are needed in ESRD patients with IE to determine appropriate indications for surgery in this high-risk and vulnerable population. Meanwhile, early involvement of CT surgical consultants in patients with any surgical indication and careful consideration of the risks and benefits of surgery may lead to more appropriate treatment of patients with this life-threatening infection.
